# The Relationship Between Multiple Sclerosis and Obesity: A Comparative Study

**DOI:** 10.7759/cureus.78530

**Published:** 2025-02-04

**Authors:** Sajad H Salih, Ali R Hashim, Nazik H Hasrat, Hassan A Farid

**Affiliations:** 1 Medicine, Basrah Teaching Hospital/Basrah Health Directorate, Basrah, IRQ; 2 Medicine, College of Medicine/University of Basrah, Basrah, IRQ; 3 Community Medicine, Training and Human Development Centre/Basrah Health Directorate, Basrah, IRQ; 4 Neurology, St George's University Hospital, London, GBR

**Keywords:** expanded disability status scale (edss), multiple sclerosis (ms), obesity, primary progressive multiple sclerosis, relapse, relapsing-remitting multiple sclerosis

## Abstract

Background: Multiple sclerosis (MS) is a chronic autoimmune disorder affecting the central nervous system. It is characterized by inflammation, demyelination, and destruction of the axons. Obesity is a worldwide health issue that involves excessive accumulation of body fat, resulting in negative metabolic and health effects. The association between MS and obesity has received more attention in recent years.

Objectives: The current study aims to evaluate the relationship between MS and obesity and determine whether obesity is a risk factor for MS development, relapse, and disability.

Methods: A case-control study was conducted at the Basrah Teaching Hospital MS clinic to compare 80 MS cases with 100 healthy controls, who were age- and sex-matched. Data collected from 1/6/2023 to 1/1/2024 includes sociodemographic factors, clinical characteristics, anthropometric measures, family and childhood obesity history, relapse frequency, and expanded disability status scale scores (EDSS).

Results: The study compared 80 MS cases with 100 controls. While mean age differed slightly between MS cases (32.34 ± 9.28 years) and controls (30.58 ± 8.67 years), it was not significant (P = 0.196). Female predominance was noted in MS cases (66.3%, n = 53) versus controls (54.0%, n = 54), with no significant difference (P = 0.126). Among the MS cases, the majority exhibit relapsing-remitting MS (RRMS) (73.7%, n = 59), followed by primary progressive MS (PPMS) (16.2%, n = 13), clinically isolated syndrome (CIS) (6.3%, n = 5), and secondary progressive MS (SPMS) (3.8%, n = 3). Childhood obesity was significantly associated with MS development (26.3%, n = 21 in MS cases vs. 1.0%, n = 1 in controls, P = 0.001). Body mass index (BMI) was significantly higher in MS cases (25.04 ± 3.61) than in controls (24.75 ± 3.37, P = 0.016), with a higher proportion of obesity (15.0%, n = 12 in MS cases vs. 5.0%, n = 5 in controls, P = 0.027). A moderate positive correlation existed between BMI and both relapse frequency (r = 0.610) and EDSS scores (r = 0.454), both statistically significant (P = 0.001).

Conclusions: The study found a significant association between increased BMI and MS occurrence, with strong associations to relapse frequency and EDSS scores. Childhood obesity was also linked to MS development, but not a family history of obesity. However, BMI alone may not reliably indicate an MS pattern.

## Introduction

Multiple sclerosis (MS) is a chronic autoimmune disorder affecting the brain and spinal cord. It is characterized by inflammation, demyelination, and destruction of the axons [1]. It has a global impact on more than 2.8 million people, mostly young adults, and is a major contributor to neurological impairment in this age group [2]. MS is characterized by a diverse array of symptoms, which include exhaustion, muscular weakness, difficulties with coordination, disruptions in sensory perception, and cognitive impairment [1]. The precise cause of MS is still unknown, despite thorough investigation. It is believed that a combination of genetic predisposition, environmental variables, and immunological dysregulation contributes to the development of MS [3].

Obesity is a worldwide health issue that involves excessive accumulation of body fat, resulting in negative metabolic and health effects. The prevalence of obesity has significantly increased in the last decades, impacting people of all age groups, socioeconomic backgrounds, and geographical areas [4].

The association between MS and obesity has received more attention in recent years. Research suggests possible interactions between these two illnesses [5]. Studies in epidemiology have shown an increased prevalence of obesity in patients with MS compared to the entire population [6]. Moreover, there is an association between obesity throughout adolescence or early adulthood and a heightened likelihood of having MS in later stages of life [7]. It is found that obesity throughout early childhood and adolescence greatly increases the likelihood of developing MS. Hence, the increasing prevalence of obesity may be a possible factor in the increased rates of MS in both children and adults [8].

Growing evidence indicates that obesity may also impact the likelihood of experiencing a relapse of MS and the development of disability [5]. There is an association between obesity and an elevated likelihood of having a relapse of MS. A higher BMI is linked to more frequent and severe relapses [5]. Moreover, obesity has been linked to the development of disability in patients with MS [6]. Research found that having a high BMI and being obese throughout youth or early adulthood is linked to faster disability development and more severe neurological impairments as time goes on [5].

Furthermore, there is compelling evidence indicating that obesity interacts with genetic and environmental variables, hence increasing the vulnerability to MS. The research analyzed data from case-control studies conducted in the United States and Sweden. It found that individuals with a BMI of 27 kg/m^2^ or higher in their early adulthood and having 1-2 risk alleles of human leukocyte antigen (HLA)-DRB1*15 had a seven-fold higher chance of developing MS compared to those who did not contain these risk alleles and had a BMI lower than 27 kg/m^2^ [9]. A significant association was identified in both populations between having a BMI of 27 kg/m^2^ or higher in early adulthood and harboring 1-2 risk alleles of HLA-A*02. This study presents compelling evidence that obesity interacts with well-established genetic risk loci for multiple sclerosis [9]. A distinct study documented a significant relationship between the BMI of adolescents and infectious mononucleosis (IM), which may be triggered by the Epstein-Barr virus and linked to an increased risk of MS. Persons with a BMI greater than 27 and a history of IM had a risk of developing MS that was more than six times higher than persons with a BMI less than 27 and without IM. Interestingly, this was much higher than the risk of MS based just on BMI [10].

The precise biological mechanism between obesity and MS remains unclear, while multiple hypotheses have been suggested. Obesity is defined by a chronic, low-grade inflammatory reaction, and the combination of metabolic tissue and immune cells contributes to obesity and the inflammation associated with obesity by sharing a common cellular target. Adipose tissue inflammation may manifest in childhood. Childhood and adolescent obesity are linked to elevated levels of C-reactive protein, interleukin-6, and leptin, indicating a state of inflammation that might impact the development of MS. Indeed, the concentrations of certain adipokines, such as leptin, adiponectin, and resistin have been seen to correlate with autoimmune disorders, including MS. Therefore, those who are overweight or obese may have a much higher likelihood of acquiring MS in comparison to individuals who have a normal weight [8].

The association between MS and obesity is complex since obesity has an impact on the likelihood of developing the illness, its progression, and the clinical results for individuals with MS [5]. The current study aims to evaluate the relationship between MS and obesity and determine whether obesity is a risk factor for MS development, relapse, and disability.

## Materials and methods

This study employs a case-control design conducted at the Basrah Teaching Hospital Multiple Sclerosis clinic, comparing individuals diagnosed with MS against a control group of healthy individuals. Data was collected from 1/6/2023 to 1/1/2024. The current study involved 180 participants, 80 of whom are MS cases who were presented to the MS clinic, and 100 controls whose age and gender were matched with the cases and collected randomly from the outpatient department, and they have no known diagnosis of neurological disorder. Consent for treatment and open access publication was obtained or waived by all participants in this study. University of Basrah Ethical Committee and the ethical committee of the Basrah Health Directorate issued approval (Approval number: 25376).

A pre-prepared data collection sheet was used to gather the data, which included sociodemographic data including age, gender, residency, smoking status, and chronic medical illnesses, and was collected through interviews and medical records reviews. It also included the clinical characteristics, including the pattern of MS (relapsing-remitting MS (RRMS), primary progressive MS (PPMS), secondary progressive MS (SPMS), and clinically isolated syndrome (CIS)) [11], age at diagnosis, clinical features, and current disease-modifying therapy (DMT), and these data were obtained from medical records and clinical review. Furthermore, the research assessed the anthropometric measures, including height, weight, and BMI, which were measured using standardized techniques and the BMI classification followed the CDC criteria [12] as follows: (1) BMI is less than 18.5, which falls within the underweight range; (2) BMI is 18.5 to <25; it falls within the healthy weight range; (3) BMI is 25.0 to <30; it falls within the overweight range; (4) BMI is 30.0 or higher; it falls within the obesity range. The researcher also gathered information about the history of obesity, which includes family and childhood obesity, which are either self-reported or obtained from medical records.

The data collection sheet also includes the frequency of relapse, which is classified into several categories based on the number of relapses experienced within a specific period [11]. These classifications typically include (1) no relapse, which indicates a period without any new or worsening symptoms characteristic of an MS relapse; (2) low frequency, which refers to experiencing one relapse within a defined timeframe; (3) moderate frequency, which involves experiencing up to two relapses within a specified timeframe; (4) high frequency, which indicates experiencing three or more relapses within a given timeframe.

Furthermore, the research used the expanded disability status scale scores (EDSS), which is a widely used measure to assess disability in individuals with MS. It categorizes disability into different levels based on the extent of neurological impairment. The classification of EDSS scores typically includes the following categories [13]: (1) EDSS 0.0-3.5, which is equivalent to minimal to no disability, and individuals who fall into this category can fully carry out all activities of daily living without significant limitations. (2) EDSS 4.0-6.5, which reflects moderate disability, and individuals in this category may experience limitations in activities of daily living, such as walking ability, but are still largely independent with varying degrees of impairment. (3) EDSS 7.0-9.5, which reflects severe disability, and individuals who fall into this category have significant limitations in mobility and may require assistance with daily activities. Wheelchair use may be common in this range. (4) EDSS 10.0, which reflects death due to MS-related complications. This score indicates the most severe level of disability, where individuals are unable to carry out any activities and are often bedridden or in a vegetative state.

The study results were analyzed using the computerized Statistical Package for Social Science (SPSS) version 26 (Armonk, NY: IBM Corp.) software. The descriptive statistics used include mean, standard deviation, frequency, and percentage; the inferential statistical methods used include the chi-square test for qualitative data, the t-test for quantitative data, and the Pearson correlation test for correlation analysis. The significance level is set at p < 0.05.

## Results

Table 1 presents the sociodemographic characteristics of 80 MS cases and 100 controls. The mean age of MS cases is 32.34 years (SD = 9.28), slightly higher than that of controls at 30.58 years (SD = 8.67), but this difference is not significant (P = 0.196). The gender distribution shows a higher proportion of females among MS cases (66.3%, n = 53) compared to controls (54.0%, n = 54), but this difference is not significant (P = 0.126). Additionally, a larger percentage of MS cases reside in urban areas (87.5%, n = 70) compared to controls (78.0%, n = 78); however, the difference is not significant (P = 0.118). Smoking status reveals that 20.0% (n = 16) of MS cases are smokers, while 26.0% (n = 26) of controls are smokers, though this difference is not statistically significant (P = 0.379). Furthermore, chronic medical illnesses are present in 2.5% of MS cases (n = 2) who are hypertensive compared to none in controls, though this difference is not statistically significant (P = 0.196).

**Table 1:** The sociodemographic characteristics of MS cases and controls SD: Standard deviation; MS: Multiple sclerosis

Characteristics		MS Cases (n = 80)	Controls (n = 100)	P-Value
Age	Mean ± SD	32.34 ± 9.28	30.58 ± 8.67	0.196
Gender	Males	27 (33.8%)	46 (46.0%)	0.126
	Females	53 (66.3%)	54 (54.0%)	
Residency	Urban	70 (87.5%)	78 (78.0%)	0.118
	Rural	10 (12.5%)	22 (22.0%)	
Smoking status	Smoker	16 (20.0%)	26 (26.0%)	0.379
	Non-smoker	64 (80.0%)	74 (74.0%)	
Chronic medical illnesses	Present	2 (2.5%)	0 (0.0%)	0.196
	Absent	78 (97.5%)	100 (100.0%)	

Table 2 provides an overview of the clinical characteristics of MS cases. Among the MS cases, the majority exhibit RRMS (73.7%, n = 59), followed by PPMS (16.2%, n = 13), CIS (6.3%, n = 5), and SPMS (3.8%, n = 3). The mean age at diagnosis is 28.04 years (SD = 7.66). Clinical features observed in MS cases include sensory symptoms (92.5%, n = 74), limb weakness (87.5%, n = 70), optic neuritis (61.3%, n = 49), and sphincter dysfunction (55.0%, n = 44). Furthermore, the distribution of current DMT among MS cases includes interferon-B (63.8%, n = 51), fingolimod (22.5%, n = 18), natalizumab (23.8%, n = 19), ocrelizumab (15.0%, n = 12), and rituximab (1.25%, n =1).

**Table 2:** The clinical characteristics of MS cases SD: Standard deviation; MS: Multiple sclerosis; RRMS: Relapsing-remitting multiple sclerosis; CIS: Clinically isolated syndrome; SPMS: Secondary progressive multiple sclerosis; DMT: Disease-modifying therapies

MS Characteristics		Frequency	Percentage
Pattern of MS	RRMS	59	73.7
	PPMS	13	16.2
	CIS	5	6.3
	SPMS	3	3.8
Age at diagnosis	Mean ± SD	28.04 ± 7.66	
Clinical features	Optic neuritis	49	61.3
	Double vision	12	15
	Nystagmus	4	5
	Gait ataxia	23	28.8
	Limb incoordination	21	26.3
	Limb weakness	70	87.5
	Sensory symptoms	74	92.5
	Sphincter dysfunction	44	55.0
	Cognitive impairment	1	1.3
	Speech problems	6	7.5
	Bowel symptoms	7	8.8
	Swallowing problems	5	6.3
Current DMT	Interferon-B	51	63.8
	Fingolimod	18	22.5
	Natalizumab	19	23.8
	Ocrelizumab	12	15.0
	Rituximab	1	1.25

Table 3 presents the association between anthropometric measures and the development of MS. The mean weight of MS cases is 68.79 kg (SD = 10.93), slightly higher than controls at 67.61 kg (SD = 12.15), although this difference is not statistically significant (p = 0.493). Similarly, the mean height of MS cases (1.63 m ± 0.08) is comparable to controls (1.65 m ± 0.08), with no significant difference observed (p = 0.182). However, the mean BMI of MS cases (25.04 ± 3.61) is significantly higher than controls (24.75 ± 3.37), with a p-value of 0.016. Further analysis of BMI grades reveals a higher proportion of obese individuals among MS cases (15.0%, n = 12) compared to controls (5.0%, n = 5) and a higher proportion of overweight individuals among MS cases (46.0%, n = 37) compared to controls (39.0%, n = 39), with a statistically significant P-value of 0.027.

**Table 3:** The association between the anthropometric measures and the development of MS SD: Standard deviation; MS: Multiple sclerosis; BMI: Body mass index

Anthropometric Measures		MS Cases (n = 80)	Controls (n = 100)	P-Value
Weight		68.79 ± 10.93	67.61 ± 12.15	0.493
Height		1.63 ± 0.08	1.65 ± 0.08	0.182
BMI	Mean ± SD	25.04 ± 3.61	24.75 ± 3.37	0.016
BMI grades	Obese	12 (15.0%)	5 (5.0%)	0.027
	Overweight	37 (46.3%)	39 (39.0%)	
	Normal	30 (37.4%)	55 (55.0%)	
	Underweight	1 (1.3%)	1 (1.0%)	

Table 4 examines the association between familial and childhood obesity and the development of MS. Regarding the family history of obesity, 6.25% (n = 5) of the MS cases have a family history of obesity in comparison with 7.0%(n = 7) of the controls, indicating no significant association (P = 0.841). Conversely, childhood obesity shows a notable association with MS development. Among MS cases, 26.3% (n = 21) report a history of childhood obesity, whereas only 1.0% (n = 1) of controls report the same. This discrepancy is statistically significant, with a P-value of 0.001.

**Table 4:** The association between familiar and childhood obesity and the development of MS MS: Multiple sclerosis

History of Obesity		MS Cases (n = 80)	Controls (n = 100)	P-Value
Family history of obesity	Present	5 (6.25%)	7 (7.0%)	0.841
	Absent	75 (93.75%)	93 (93%)	
Childhood obesity	Present	21 (26.3%)	1 (1.0%)	0.001
	Absent	59 (73.7%)	99 (99.0%)	

Table 5 illustrates the association between BMI and the pattern of MS. The mean BMI varies across MS patterns, with PPMS cases having the highest mean BMI at 27.85 kg/m² (SD = 5.44), followed by CIS (26.60 kg/m², SD = 2.32), SPMS (26.16 kg/m², SD = 4.37) and RRMS (25.72 kg/m², SD = 3.18), although this difference is not statistically significant (p = 0.566). However, analysis of BMI grades reveals significant differences among MS patterns. Specifically, a higher proportion of obese individuals is observed in PPMS (38.5%, n = 5) compared to CIS (20.0%, n = 1) and RRMS (8.5%, n = 5), with a statistically significant P-value of 0.005. Conversely, RRMS cases exhibit the highest proportion of overweight individuals (50.8%, n = 30), while SPMS cases have the highest proportion of normal BMI (66.7%, n = 2).

**Table 5:** The association between BMI and the pattern of MS SD: Standard deviation; MS: Multiple sclerosis; RRMS: Relapsing-remitting multiple sclerosis; CIS: Clinically isolated syndrome; SPMS: Secondary progressive multiple sclerosis; BMI: Body mass index

Anthropometric Measures		CIS (n = 5)	PPMS (n = 13)	RRMS (n = 59)	SPMS (n = 3)	P-Value
BMI	Mean ± SD	26.60 ± 2.32	27.85 ± 5.44	25.72 ± 3.18	26.16 ± 4.37	0.566
BMI grades	Obese	1 (20.0%)	5 (38.5%)	5 (8.5%)	1 (33.3%)	0.005
	Overweight	4 (80.0%)	3 (23.1%)	30 (50.8%)	0 (0.0%)	
	Normal	0 (0.0%)	4 (30.7%)	24 (40.7%)	2 (66.7%)	
	Underweight	0 (0.0%)	1 (7.7%)	0 (0.0%)	0 (0.0%)	

Table 6 investigates the association between BMI and the frequency of relapse. The mean BMI varies significantly across relapse frequency groups, with individuals experiencing no relapse having the lowest mean BMI (23.45 kg/m², SD = 1.86), followed by those with low frequency (24.72 kg/m², SD = 3.34) and moderate frequency (25.71 kg/m², SD = 2.19) of relapse. Notably, individuals with high-frequency relapses exhibit the highest mean BMI (28.97 kg/m², SD = 4.08), demonstrating a statistically significant difference (p = 0.001). Further analysis of BMI grades reveals a notable association between obesity and relapse frequency. Specifically, 50.0% (n = 11) of individuals with high-frequency relapses are obese, compared to none in the no-relapse group, indicating a significant relationship with a p-value of 0.001. Conversely, the majority of individuals with no relapse have a normal BMI (72.7%, n = 8), while those with high-frequency relapses have the highest proportion of overweight individuals (61.5%, n = 16).

**Table 6:** The association between BMI and frequency of relapse BMI: Body mass index, SD: Standard deviation

Anthropometric Measures		No Relapse (n = 11)	Low Frequency (1) (n = 21)	Moderate Frequency (≤ 2) (n = 26)	High Frequency (≥ 3) (n = 22)	P-Value
BMI	Mean ± SD	23.45 ± 1.86	24.72 ± 3.34	25.71 ± 2.19	28.97 ± 4.08	0.001
BMI grades	Obese	0 (0.0%)	1 (4.8%)	0 (0.0%)	11 (50.0%)	0.001
	Overweight	3 (27.3%)	9 (42.8%)	16 (61.5%)	9 (40.9%)	
	Normal	8 (72.7%)	10 (47.6%)	10 (38.5%)	2 (9.1%)	
	Underweight	0 (0.0%)	1 (4.8%)	0 (0.0%)	0 (0.0%)	

Table 7 examines the association between BMI and EDSS scores. The mean BMI varies significantly across disability levels, with individuals experiencing low disability having the lowest mean BMI (25.05 kg/m², SD = 2.79), followed by those with moderate disability (25.96 kg/m², SD = 3.41) and high disability (29.84 kg/m², SD = 4.20). This difference is statistically significant (p = 0.001). Further analysis of BMI grades reveals a significant association between obesity and disability level. Specifically, 71.4% (n = 10) of individuals with a high disability are obese, compared to 1.9% (n = 1) in the low disability group, with a p-value of 0.001. Conversely, the majority of individuals with low disability have a normal BMI (45.3%, n = 24), while those with moderate disability exhibit a higher proportion of overweight individuals (61.5%, n = 8).

**Table 7:** The association between BMI and EDSS SD: Standard deviation; BMI: Body mass index; EDSS: Expanded Disability Status Scale

Anthropometric Measures		Low Disability (EDSS 0-3.5) (n = 53)	Moderate Disability (EDSS 4.0-6.5) (n = 13)	High Disability (EDSS 7.0-9.5) (n= 14)	Death (EDSS 10.0) (n = 0)	P-Value
BMI	Mean ± SD	25.05 ± 2.79	25.96 ± 3.41	29.84 ± 4.20	-	0.001
BMI grades	Obese	1 (1.9%)	1 (7.7%)	10 (71.4%)	0 (0.0%)	0.001
	Overweight	27 (50.9%)	8 (61.5%)	2 (14.3%)	0 (0.0%)	
	Normal	24 (45.3%)	4 (30.8%)	2 (14.3%)	0 (0.0%)	
	Underweight	1 (1.9%)	0 (0.0%)	0 (0.0%)	0 (0.0%)	

**Figure 1:**
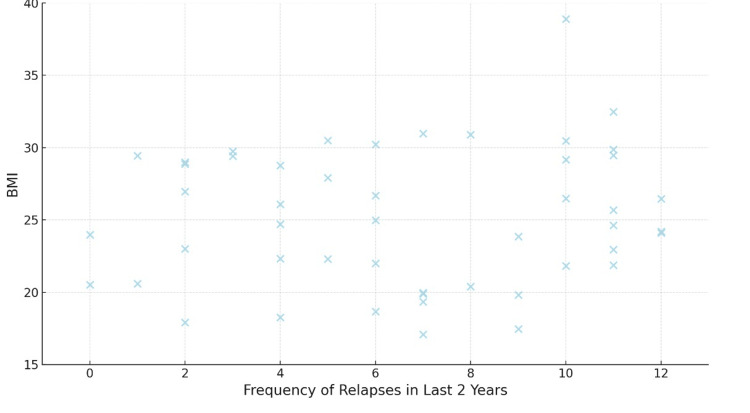
The correlation between BMI and the frequency of relapse BMI: Body mass index The image was created by the authors of this article.

**Figure 2:**
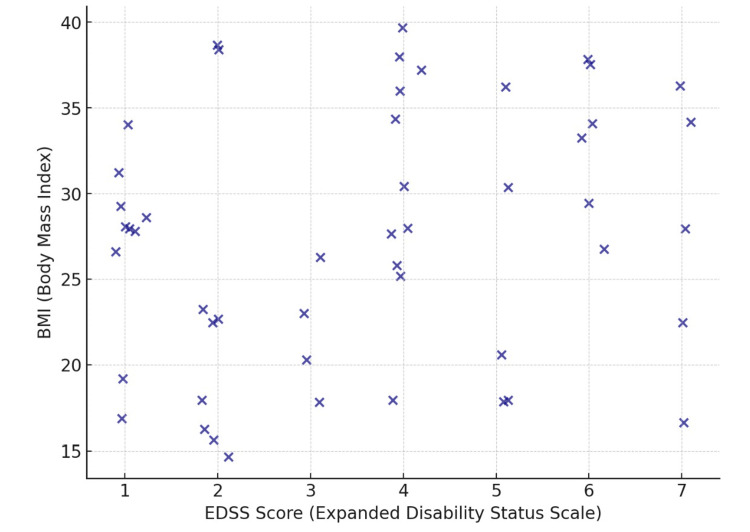
The correlation of BMI with EDSS BMI: Body mass index, EDSS: Expanded Disability Status Scale The image was created by the authors of this article.

Table 8 presents the correlation between BMI and both the frequency of relapse and the EDSS scores. The correlation coefficient (r-value) between BMI and the frequency of relapse is 0.610, indicating a moderate positive correlation between higher BMI and increased frequency of relapse in individuals with MS (Figure 1). This correlation is statistically significant with a p-value of 0.001, suggesting that BMI is significantly associated with the frequency of relapse. Similarly, the correlation coefficient between BMI and EDSS scores is 0.454, indicating a moderate positive correlation between higher BMI and increased disability as measured by the EDSS (Figure 2). This correlation is also statistically significant with a p-value of 0.001, indicating that BMI is significantly associated with disability severity in individuals with MS.

**Table 8:** The correlation of BMI with the frequency of relapse and EDDS BMI: Body mass index; EDSS: Expanded Disability Status Scale

Correlation		Frequency of Relapse	EDSS
BMI	r-value	0.610	0.454
	P-value	0.001	0.001

## Discussion

The correlation between MS and obesity has received more attention in recent years. Studies indicate that obesity might be a contributing factor in the development of multiple sclerosis and can worsen its symptoms, possibly resulting in more severe consequences. Comprehending this correlation is essential due to the ongoing increase in obesity rates worldwide, which is causing apprehension over its effects on public health [14]. Examining the correlation between multiple sclerosis and obesity might provide an essential understanding of the fundamental processes of the illness, perhaps resulting in novel approaches for preventing and controlling it. Furthermore, tackling obesity might provide possibilities to enhance the general well-being and standard of living for those who have MS.

The present research examined instances with MS in comparison to a control group. Matching was conducted to reduce the influence of confounding factors, as shown in Table 1. The age distribution of MS patients and controls does not exhibit a statistically significant difference (p = 0.196), suggesting that the age matching was effective. The gender distribution between male and female participants in both groups was evenly balanced, and no statistically significant difference was found (p = 0.126). With respect to place of residence, there was a higher proportion of urban inhabitants among individuals with MS compared to the control group. However, this difference did not reach statistical significance (p = 0.118). There were no significant differences in smoking status and the existence of chronic medical diseases between cases and controls (p = 0.379 and p = 0.196, respectively). These results indicate that the matched groups possess comparable demographic and lifestyle features, which decreases the probability of extraneous variables affecting the observed connections between MS and the examined obesity determinants.

In this research, the occurrence of RRMS represents 73.7% of the cases, which is significantly less than the expected 85% reported by Thompson et al. [15]. The disparity might be ascribed to differences in the demographics and research approaches of the study groups. The present research reports a prevalence of 16.2% for PPMS, which closely corresponds to the expected range of 10-15% indicated by Lublin et al. [16]. The consistent nature of PPMS frequency across many investigations indicates its relative stability. Clinically isolated syndrome accounts for 6.3% of cases, a figure that falls within the range of 20-30% reported by Thompson et al. [15]. This implies that the prevalence of CIS in the research population may be lower compared to other cohorts. The prevalence of secondary progressive MS is 3.8%, which is much lower than the expected transition rate of 50% from RRMS to SPMS during a period of 10-20 years, as reported by Lubin et al. [16]. This may suggest variations in the advancement of the illness or the criteria for selecting participants in the present research. It is worth noting that the majority of our patients have been recently diagnosed with MS, as shown in Table 2.

The present investigation demonstrates that the observed clinical characteristics in individuals with MS, as shown in Table 2, are mostly in agreement with the results reported in previous research. In research conducted by Kister et al. [17], it was shown that optic neuritis occurred in about 50-60% of MS cases. Limb weakness was identified in 80-90% of cases, while sensory abnormalities were widespread in over 90% of patients. Likewise, there was a high occurrence of gait ataxia and sphincter dysfunction, which is consistent with the percentages shown in our research (Table 2). Nevertheless, discrepancies across studies might arise as a result of variables such as the size of the sample, the geographical location, and the technique used. Alroughani et al. [18] conducted research that revealed a greater occurrence of cognitive impairment in individuals with MS as compared to the proportion shown in the table. The discrepancies in evaluation methodologies, illness duration, and patient groups might be the underlying causes of these variances.

The results shown in Table 3 on the correlation between anthropometric measurements and the development of MS are consistent with various prior investigations. For example, research conducted by Munger et al. [19] discovered that individuals with a higher BMI during their early adulthood had an increased likelihood of acquiring MS later in their lives. In a separate investigation conducted by Cortese et al. [20], it was shown that individuals with a BMI higher than 25 kg/m^2^ had a considerably greater chance of developing MS. Similarly, research conducted by Hedström et al. [21] discovered that those who had a BMI over 27 kg/m^2^ at the age of 20 had double the chance of having MS compared to individuals with a normal weight. The studies provide evidence that having a high BMI, especially during important stages like early adulthood, can contribute to the development of MS. We also found that among people with MS, 26.3% have a history of childhood obesity, while only 1.0% of the control group reported the same (Table 4). The consistent results from these studies highlight the significance of tackling obesity as a modifiable risk factor for MS via tailored therapies focused on managing weight and making lifestyle changes. Conversely, Gianfrancesco et al. [22] conducted research to investigate the correlation between higher BMI and MS. Comparable findings were discovered, indicating that girls with a BMI > 30 kg/m^2^ had a twofold higher chance of developing MS. However, no significant correlation was seen in men. In contrast to previous results, new research conducted by Hedström et al. [23] reveals a higher susceptibility to obesity in adolescents, but not in children. Nevertheless, it is crucial to focus on addressing obesity in infancy as a means of decreasing the likelihood of developing MS in the general population. According to a study conducted by Ascherio and Munger [24], it has been projected that if childhood obesity is eliminated, over 15% of occurrences of MS might be prevented.

The link between obesity and the distinct forms of MS, such as progressive or relapse-remitting, is now being actively researched in the field of neurology. Although research has offered some understanding of how obesity affects the chance of developing multiple sclerosis and the course of the illness, the connection between obesity and certain forms of MS is intricate and not completely understood. Several studies indicate that obesity may be a contributing factor to an increased susceptibility to developing MS, as well as impacting the progression of the illness. For example, a comprehensive analysis conducted by Langer-Gould et al. [5] found that obese adults had a slightly higher likelihood of acquiring MS. Nevertheless, the impact of obesity on the advancement and distinct manifestations of MS, such as relapse-remitting or progressive variants, remains uncertain. Recent data indicates possible connections between inflammation caused by obesity, metabolic dysfunction, and neuroinflammatory processes involved in the development of MS. However, more research using extensive long-term studies and improved imaging methods is necessary to clarify the exact pathways that link obesity with certain patterns of MS. Gaining a comprehensive understanding of these connections may provide significant knowledge for developing personalized treatment approaches and treatments to reduce the effects of obesity on MS outcomes.

The association between obesity, disability (as shown by a high EDSS score), and the risk of death is a significant subject for specialists who treat patients with MS. Obesity was shown to be correlated with a more rapid rise in scores on the EDSS as compared to those with normal weight. According to Pinhas-Hamiel et al. [25], persons with multiple sclerosis with a considerable level of impairment (EDSS score ≥ 3.0) had a 1.7-fold higher likelihood of being overweight and obese compared to controls of the same age and gender. Similarly, a comprehensive investigation conducted by Fitzgerald et al. [26] revealed a significant correlation between higher levels of central obesity and serious disability in persons with MS. In addition, further research conducted by Mowry et al. [27] found that there was a strong correlation between greater BMI and increased EDSS scores. Specifically, for every 5 kg/m^2^ rise in BMI, the EDSS score was 0.13 points higher. The results of these research studies are consistent with our findings. Conversely, recent research conducted by Livne-Margolin et al. [28] revealed that whereas central obesity is linked to impairment in persons with MS, BMI is not. When considering persons with disabilities, BMI is not a reliable measure of body fat since disability may cause alterations in the way fat is distributed and the overall composition of the body. Diminished muscular activity causes muscle volume reduction and atrophy, resulting in a decrease in BMI. Simultaneously, the build-up of fat in the abdominal area leads to an increase in waist circumference. Moreover, research conducted by Livne-Margolin et al. [28] revealed that persons who have a normal BMI but also have central obesity have a greater mortality rate compared to overweight or obese individuals. This suggests that central obesity increases the risk of mortality.

The current study was done at a single center, which might restrict the applicability of the results to more extensive populations. Despite the inclusion of 180 individuals in the study, the sample size may still be considered somewhat small, which may have an impact on the statistical power and accuracy of the findings. The control group was chosen from the outpatient department, potentially leading to selection bias since these people may not truly reflect the broader population. We collected data on childhood and familial obesity history, clinical presentation, and relapse frequency from self-reporting or medical records, potentially subject to recall or data-completion bias. The research may not have included all possible confounding factors that may affect the observed relationships, such as food patterns, levels of physical activity, and socioeconomic status. Furthermore, the absence of comprehensive information from medical records and the paucity of MRI reports contribute to the insufficient information on disease activity on MRI.

## Conclusions

The research identified a significant association between increased BMI and the occurrence of multiple sclerosis, suggesting that obesity might potentially be a contributing factor to the development of MS. There was a strong association between BMI and both the frequency of relapse and EDSS scores, indicating that higher BMI is linked to greater disease severity and impairment in patients with MS. Childhood obesity was shown to have an important relationship with the development of MS, although no significant correlation was observed between a family history of obesity and MS. Considering the link between childhood obesity and the onset of MS, implementing early intervention programs that focus on preventing and controlling obesity throughout childhood may potentially decrease the likelihood of developing MS in the future. Efforts to address obesity in public health might have substantial consequences for the prevention of MS. It is also worth recommending performing intervention studies to assess the effects of weight management therapies on MS outcomes, including the incidence of relapses and the development of disability.
